# Assessment of the Relationship between Physical Working Conditions and Different Levels of Work Ability

**DOI:** 10.5539/gjhs.v6n4p213

**Published:** 2014-04-20

**Authors:** Mirsaeed Attarchi, Mostafa Ghaffari, Alireza Abdi, Elham Mirzamohammadi, Seyed Mohammad Seyedmehdi, Farzaneh Rahimpour, Maryam Fazlalizadeh, Saber Mohammadi

**Affiliations:** 1 Brain and Spinal Cord Injury Research Center (BASIR), Imam Khomeini Hospital, Tehran University of Medical Sciences, Tehran, Iran; 2 Occupational Medicine Department, School of Medicine, Iran University of Medical Science, Tehran, Iran; 3 National Research Institute of Tuberculosis and Lung Disease, Shahid Beheshti University of Medical Sciences, Tehran, Iran; 4 Occupational Medicine Department, School of Medicine, Mashhad University of Medical Sciences, Mashhad, Iran

**Keywords:** workability, physical working conditions, worker

## Abstract

Early leaving of workplace by work forces is one of the fundamental problems worldwide. Maintenance and enhancement of employees work ability are important for raising productivity.

This study investigated the relationship between work ability index and physical working conditions and was carried out in 2013 on 641 workers at a manufacturing plant in Tehran. Work ability was assessed by the questionnaire of work ability index and the participants were classified into four work ability groups of poor, moderate, good, and excellent. Physical working conditions were evaluated by the MUSIC-Norrtalje questionnaire and the participants were classified into two groups with proper and poor physical working conditions. The mean score of work ability questionnaire was 42.40; and 2.5% (16 persons), 9.2% (59 persons), 38.2% (245 persons), and 50.1% (321 persons) of the participants were in poor, moderate, good, and excellent work ability groups, respectively. The mean score of physical working conditions questionnaire was 20.06. The results of logistic regression analysis showed that even after adjusting the confounding variables, a significant correlation existed between work ability and physical working conditions (p<0.05). According to the results of this study, there may be a correlation between physical working conditions such as awkward postures, repetitive movements, load lifting, exposure to whole body vibration and so on with work ability. Therefore it seems that enhancement of the quality of physical working conditions may increase work ability.

## 1. Introduction

Early leaving of workplace by work forces is one of the fundamental problems in developed and developing countries (Ilmarinen, 2001). Maintenance and enhancement of employees work ability are important for raising productivity and preventing them from early leaving of workplace. Proper work ability, especially in jobs with high demands, is considered as a prerequisite to cope with job requirements in a variety of working conditions. Therefore, assessment of work ability and identifying individuals with inappropriate work performance will gain importance more than ever in the near future.

Work ability is defined as the amount of job that a worker can perform at present and future, and how he/she will do his/her work according to job requirements, personal health status, and mind resources (Lin et al., 2006).

Based on previous studies, the major factors affecting work ability include occupational factors (psychological and physical factors), health status, lifestyle, and physical activities. Imbalance between psychological and physical capacities of workers and job requirements has increasingly resulted in workers’ health problems and ultimately has led to work force replacement and work force loss (Stattin, 2005).

In a systematic review, 14 cross-sectional and 6 longitudinal studies were assessed and high physical workload and poor physical work environment were reported as the important factors decreasing work ability. A relationship was seen between physical activity during leisure time, musculoskeletal capacity, age, obesity, and mental demand of work with work ability (van den Berg et al., 2009). In previous studies, high physical demands such as heavy muscular work, poor working posture, and poor ergonomic conditions were significantly related to work ability index (Pohjonen, 2001; Tuomi et al., 1991; Tuomi et al., 1997; Tuomi et al., 2004). In previous studies, reported an inverse relationship between work ability index and thermal discomfort and poor physical climate (Tuomi et al., 1991; Tuomi et al., 2007). However, this association was not observed in some other studies (Fischer et al., 2006; Tuomi et al., 1997).

In a cross-sectional study, Martinez et al. (2006) analyzed 224 employees in Sao Polo. Their results showed that all the studied aspects of health were related to work ability. They found also an association between work ability and demographic and occupational factors.

In a cross-sectional study on 19,507 Dutch construction workers in 2005, Alavinia et al. (2007) investigated the occupational and personal factors affecting work ability. The results of this study showed that physical workload and with a lesser extent, psychological factors affected work ability. One of the most important factors in this study which affected work ability was ergonomic risk factors; moreover, poor posture, static posture, and repetitive movements had the highest correlation with work ability.

The association of physical working conditions with work ability was less considered in previous studies. Workers in automotive sub-industry are facing with various physical risk factors and on the other hand, few studies was performed in this field in Iran, thus the present study aimed to assess work ability with the questionnaire of work ability index and determine its association with physical working conditions in an automobile seat factory.

## 2. Method

### 2.1 Study Design and Population

This study was conducted at a manufacturing industrial company located in Tehran in 2013. All the male workers of the plant were enrolled in the study. From among 931 production line workers, 641 consented to cooperate and participated in the study (Response Rate=68.85%). The participants’ demographic (including age, education level, marital status, cigarette smoking etc.), medical (history of disease, medication etc.), and occupational (work experience, work shift etc.) information were collected using direct interviews. The participants’ duration of leave of absence was obtained through the Administrative Affairs Department in the relevant plant. Moreover, the participants’ weight and height were measured and the Body Mass Index (BMI) was calculated in kg/m^2^. All workers participated voluntarily in this study and signed informed consent form. This study was approved by the ethics committee of Tehran University of medical sciences.

### 2.2 Work Ability Measurement

The participants’ work ability was assessed using the Work Ability Index questionnaire (Tuomi et al., 1994). The Persian version of this questionnaire, which its reliability and validity have been identified, has been used in this study (Abdolalizadeh et al., 2012). The questionnaire consists of 7 items, each with a different score range. The first item is “current work ability compared with the lifetime best’, ranging from 0 to 10 scores. The second item is “work ability in relation to job demands”, ranging from 2 to 10 scores. The third item is “number of current diseases diagnosed by a physician”, ranging from 1 to 7 scores. The fourth item is “estimated work impairment due to diseases”, ranging from 1 to 6 scores. The fifth item is “sick leave during the past year”, ranging from 1 to 5 scores. The sixth item is “prognosis of work ability within 2 recent years”, with 1, 4, or 7 scores. Finally, the seventh item is “mental resources”, ranging from 1 to 4 scores. In all the items mentioned above, the best status belongs to those with highest scores. The WAI is calculated by the sum of the scores of these 7 items. Higher WAI scores are indicative of better work ability. The scores of this index range from 7 to 49. Moreover, the participants can be classified into four work ability groups, including poor (score 7-27), moderate (score 28-36), good (score 37-43), or excellent (score 44-49) (Tuomi et al., 1994).

### 2.3 Assessment of Physical Working Conditions

Physical working conditions in this study were assessed by the Persian version of MUSIC-Norrtalje questionnaire (Alipour et al., 2007). The reliability and validity of this questionnaire have been reviewed by Alipour et al. (2007) (kappa = 0.3-0.9). The physical working conditions questionnaire consisted of 12 questions; one of them was about the total amount of work hardness with a score range of 6 for proper physical working conditions to 20 for very hard physical working conditions. The answers of other 11 questions were designed as Likert scale with 5 options as never, ¼ of the time, half the time, ¾ of the time, and all the time. These options were specified to the participants as scores 0 to 4. Higher scores of the questionnaire indicated poorer physical working conditions.

The mentioned 11 questions included 4 questions about poor posture and repetitive movements, 2 questions about load lifting, 2 questions about exposure to whole body vibration and hand-arm vibration, and 3 questions about the duration of sitting and sedentary working conditions and the duration of subtle work. In the present study, the participants were classified into two groups of proper physical working conditions and poor physical working conditions based on the median score of 11 questions of this questionnaire (22 points).

### 2.4 Statistical Analysis

Mean, standard deviation (SD) or their ranges (minimum – maximum) of quantitative variables were calculated. The independent T-test was used to compare these variables among the groups. The Chi-square was used to compare the qualitative variables. For identification of risk of moderate or lower work ability, logistic regression analysis was performed. Logistic regression and linear regression analysis with eliminating the confounding variables used to investigate the correlation between work ability and physical working conditions. P values were two-sided and less than 0.05 were considered statistically significant. The results of statistical analysis are expressed as odds ratio (OR) with 95% confidence intervals (95 %CI). All the mentioned calculations were performed using SPSS version 12 software.

## 3. Findings

In this study, 641 persons from the production line of a factory, all of whom were male, were studied. The mean age of the study population was 34.51 years with a range of 21-60 years and a mean work experience of 13.80 years with a range of 1-30 years. The mean of body mass index (BMI) of the participants was 25.60 kg/m^2^ with a range of 19.31-49.17 kg/m^2^. Two hundred and eleven persons (32.9%) had regular physical activity, 452 persons were shift workers (70.5%), and 141 persons were smokers (22.0%).

The mean score of the physical working conditions questionnaire was 20.06 with a range of 1-44.

The mean score of work ability index was 42.40 with a range of 21-49. In terms of the amount of work ability index, 16 persons (2.5%), 59 persons (9.2%), 245 persons (38.2%), and 321 persons (50.1%) were in poor, moderate, good, and excellent groups, respectively. Therefore 11.7% of the participants were in the poor or moderate groups and 88.3% in the good or excellent groups.

Table 1 compares the mean and frequency of variables in terms of work ability in different groups. Mean age, work experience, BMI, and scores of physical working conditions in the poor work ability group were higher than those in the moderate, good, and excellent work ability groups and this difference was statistically significant (*p*<0.05). Furthermore, the frequency of smokers and individuals without physical activity was significantly higher in the poor work ability group than the moderate, good, and excellent work ability groups (*p*<0.05).

**Table 1:** Comparison of the mean and frequency of the study variables in terms of work ability index group

Variable	WAI Group				P-value

	Poor(n=16)	Moderate(n=59)	Good(n=245)	Excellent(n=321)	
**The physical working conditions** (score) - Mean (SD)	36.43 (12.57)	30.50 (10.84)	26.41(11.63)	26.20 (11.53)	< 0.001
**Age** (year) - Mean (SD)	38.12 (7.56)	35.78 (6.27)	35.08 (7.75)	33.67 (6.52)	0.007
**Work experience** (year)- Mean (SD)	16.25 (5.47)	15.08 (5.35)	14.30 (6.47)	13.05 (6.05)	0.012
**Body mass index** (kg/m^2^)- Mean(SD)	28.95 (4.62)	26.53 (5.12)	25.94 (3.67)	24.67 (2.63)	< 0.001
**Work shift** (No) N (%)	8 (50.00)	11 (18.64)	72 (29.38)	98 (30.52)	0.081
**Smoking** (No) N (%)	8 (50.00)	38 (64.40)	189 (77.14)	265 (82.55)	< 0.001
**Physical activity** (No) N (%)	15 (93.75)	50 (84.74)	172 (70.20)	193 (60.12)	< 0.001

Table 2 compares the mean work ability index in terms of the study variables including physical working conditions.

**Table 2:** Comparison of the mean work ability index in terms of the study variables

Variable	WAI			

	Status	Mean	SD	P-value
**The physical working conditions** (score)	> 30(n= 243)	41.78	6.38	0.027
	≤ 30 (n=398)	42.78	4.97	
**Age** (year)	> 33 (n= 325)	41.73	6.18	0.002
	≤ 33 (n= 316)	43.09	4.76	
**Work experience** (year)	> 12 (n= 336)	41.52	6.04	< 0.001
	≤ 12 (n= 305)	43.36	4.81	
**Work shift**	Day work (n= 189)	42.41	5.91	0.965
	Shift work (n= 452)	42.39	5.42	
**Body mass index** (kg/m^2^)	≤ 25 (n= 353)	43.70	5.04	< 0.001
	> 25 (n= 288)	40.80	5.76	
**Smoking**	Yes (n= 141)	40.87	6.02	< 0.001
	No (n= 500)	42.83	5.35	
**Physical activity**	Yes (n= 211)	43.94	4.43	< 0.001
	No (n= 430)	41.64	5.90	

As this table shows, the mean of work ability index in the group with BMI equal or less than 25 kg/m^2^ was significantly higher than the group with BMI more than 25 kg/m^2^. Similarly, the mean of work ability index in the non-smokers was significantly higher than the smokers and in the group with physical activity was significantly higher than the group without physical activity (*p*<0.001).

Moreover, the mean of work ability index in the group aged 33 years or less was significantly higher than the group aged more than 33 years. Similarly, the mean of work ability index in the group with 12 years work experience or less was significantly higher than the group with more than 12 years work experience and the mean of work ability index in the group with proper physical working conditions was significantly higher than the group with poor physical working conditions (*p*<0.05).

Table 3 compares the frequency of individuals with good or excellent work ability in terms of studied variables such as physical working conditions. The frequency of persons with good or excellent work ability in the group with BMI equal or less than 25 kg/m^2^ was significantly higher than the group with BMI more than 25 kg/m^2^. Similarly, the frequency of persons with good or excellent work ability in the non-smokers group was significantly higher than the smokers group and the frequency of persons with good or excellent work ability in the group with physical activity was significantly higher than the group without physical activity (*p*<0.05). Also, the frequency of persons with good or excellent work ability in the group aged 33 years or less was significantly higher than the group aged more than 33 years and the frequency of persons with good or excellent work ability in the group with 12 years work experience or less was significantly higher than the group with more than 12 years work experience and the frequency of persons with good or excellent work ability in the group with proper physical working conditions was significantly higher than the group with poor physical working conditions (*p*<0.05).

**Table 3:** Comparison of the frequency of workers with good or excellent work ability in terms of the studied variables

Variable	N (%)	OR	95% CI	P-value
**The physical working conditions** (score)		2.04	1.25-3.31	0.005
≤ 30 (n=398)	363 (91.20)			
>30(n= 243)	203 (83.53)			
**Age** (year)		1.98	1.19-3.27	0.009
≤ 33 (n= 316)	290 (91.77)			
> 33 (n= 325)	276 (84.92)			
**Work experience** (year)		2.40	1.42-4.06	0.001
≤ 12 (n= 305)	283 (92.78)			
> 12 (n= 336)	283 (84.22)			
**Body mass index** (kg/m^2^)		1.99	1.22-3.25	0.006
≤ 25 (n= 353)	332 (91.50)			
> 25 (n= 288)	243 (84.37)			
**Smoking**		2.55	1.53-4.25	0.001
No (n= 500)	454 (90.80)			
Yes (n= 141)	112 (79.45)			
**Physical activity**				<0.001
Yes (n= 211)	201 (95.26)	3.57	1.80-7.12	
No (n= 430)	365 (84.88)			

According to the questions about load lifting and work posture, the participants were classified into two groups of lifting loads equal or heavier than 15 kg and lighter than 15 kg and two groups of proper physical posture and poor physical posture. The frequency of the individuals with good or excellent work ability in the group with lighter than 15 kg load lifting and proper physical posture was significantly higher than the group equal or heavier than 15 kg load lifting and poor physical posture. (Heavy lifting; OR = 3.63, 95% CI = 1.13-7.52; Poor posture; OR = 2.97, 95% CI = 1.08-6.03).

Linear regression analysis and logistic regression methods were used to study more accurately the relationship of work ability and physical working conditions. The variables considered in these analyses included age, marital status, education level, BMI, smoking, physical activity, work experience, work shift, and occupational factors (physical factors by MUSIC-Norrtalje Questionnaire and psychosocial factors by the Copenhagen Psychosocial Questionnaire).

The results of linear regression analysis showed that a significant correlation existed between the scores of physical factors of the work environment and the work ability index, and that the work ability index decreased by increasing the scores of work environment physical factors questionnaire (*p*<0.001). The results of this analysis also showed a significant association between smoking, age, work experience, physical activity, and BMI with the work ability index (*p*<0.05) (Table 4).

**Table 4:** Association between work ability index and physical working conditions using linear regression analysis

Variables	β	Standard Error	P-value
**Constant**	55.98	2.10	< 0.001
**The physical working conditions** (Score)	-0.07	0.01	< 0.001
**Age** (year)	-0.11	0.05	0.030
**Work experience** (year)	-0.34	0.03	0.041
**Body mass index** (kg/m^2^)	-0.32	0.05	< 0.001
**Smoking** (pack-years)	-1.51	0.50	0.003
**Physical activity**	1.79	0.44	< 0.001

Work ability was considered as a dependent variable in linear regression analysis method and according to the scoring of the work ability questionnaire, the participants were classified into two work ability groups of poor or moderate and good or excellent. Also, according to physical working conditions scores of the MUSIC-Norrtalje questionnaire, the participants were classified into two groups of proper physical working conditions and poor physical working conditions. The results of this analysis showed that even after adjusting the effect of confounding variables, a significant association existed between work ability and physical working conditions (*p*=0.003) (Table 5). There was also a significant relationship between age, BMI, smoking, physical activity, and work experience with work ability (*p*<0.05).

**Table 5:** Association between work ability index and physical working conditions using logistic regression analysis

Variable	Status	Adjusted OR	95% C.I	P-value
**The physical working conditions** (score)	> 30	1.00	-----------	0.003
	≤ 30	2.23	1.32-3.76	
**Age** (year)	> 33	1.00	-----------	0.044
	≤ 33	2.09	1.04-3.01	
**Work experience** (year)	> 12	1.00	-----------	0.046
	≤ 12	2.07	1.02-2.96	
**Body mass index** (kg/m^2^)	≤ 25	1.00	-----------	0.029
	> 25	1.78	1.06-2.99	
**Smoking**	No	1.00	-----------	0.006
	Yes	2.12	1.24-3.62	
**Physical activity**	Yes	1.00	-----------	0.002
	No	3.13	1.54-6.35	

## 4. Discussion

In terms of occupational health management, it is important to assess the work ability index. In previous studies, the work ability index has been correlated to absenteeism and disability (Alavinia et al., 2009a; Alavinia et al., 2009b).

In the present study, the mean score of work ability index was 42.40. With a frequency of 50.1%, 38.2%, 9.2% and 2.5%, the participants were in the excellent, good, moderate, and poor work ability groups, respectively.

Kaleta et al. (2004) studied the work ability index and its association with physical activity in leisure time in Poland. The mean score of work ability index in this study was 41.4 and 42.4% of the participants were in the group with excellent work ability which is consistent with the results of our study.

In the present study, the frequency of individuals with good or excellent work ability in the group with proper physical working conditions was significantly two times greater than the group with poor physical working conditions (*p*=0.003). Also, these results revealed the association of the work ability index score with the score of physical working conditions (*p*<0.001). A correlation was observed as well between working posture and load lifting with work ability.

Previous studies had showed the relationship between repetitive movements, poor working posture, and work physical demands with work ability index (Pohjonen, 2001; Sjögren-Rönkä et al., 2002; Tuomi et al., 1991; Tuomi et al., 1997).

In a study on construction workers, work ability index and factors affecting it, such as occupational factors, were analyzed. In this study, 3.4%, 49.5%, 14.2%, and 1.9% of the workers were in groups with excellent, good, moderate, and poor work ability, respectively, which is largely consistent with our study (Alavinia et al., 2007). Also in this study, the relationship of physical working conditions characteristics with work ability index was specified. A relationship was also identified between age and work ability index that was consistent with our study.

Aittomäki et al. (2003) assessed work ability index and studied its relationship with working conditions and socioeconomic status in municipal employees over 40 years in Helsinki. Their results showed that people with lower socioeconomic status had a poorer work ability and work ability in blue-collar workers was lower than white-collar workers (men: OR = 2.35; women: OR = 1.85).

Airila et al. (2012) examined the work ability index and its relationship with age, lifestyle, and occupational factors in Finland on 403 firefighters. In this study, participants were evaluated between 1999 and 2009 in terms of working conditions, job demands, physical workload, supervisory relations, and task resources. The work ability index in this study was 33.2 and 35.2 in 1999 and 2009, respectively. Also, after adjustment the confounding variables, an association was determined between work ability index and age, physical activity, and physical workload which these results are corroborated with our study.

A significant relationship was observed in our study between work ability index and age, work experience, and life style, which is consistent with previous studies (Airila et al., 2012; Ghaddar et al., 2011; Kaleta et al., 2006; Monteiro et al., 2006).

The present study was performed cross-sectionally which is a limitation because in these types of studies the certainty of causal relationship is not entirely clear. In addition, the lack of female sex workers in this study limits the generalization of the results to the entire employees.

## 5. Conclusion

In general, the results of our study propose an association between work ability and physical working conditions. If our results are confirmed by longitudinal studies, it seems that improving of physical working conditions can lead to increment of work ability.

## References

[ref1] Abdolalizadeh M, Arastoo A. A, Ghsemzadeh R, Montazeri A, Ahmadi K, Azizi A (2012). The psychometric properties of an Iranian translation of the Work Ability Index (WAI) questionnaire. J Occup Rehabil.

[ref2] Airila A, Hakanen J, Punakallio A, Lusa S, Luukkonen R (2012). Is work engagement related to work ability beyond working conditions and lifestyle factors?. Int Arch Occup Environ Health.

[ref3] Aittomäki A, Lahelma E, Roos E (2003). Work conditions and socioeconomic inequalities in work ability. Scand J Work Environ Health.

[ref4] Alavinia S. M, van Duivenbooden C, Burdorf A (2007). Influence of work-related factors and individual characteristics on work ability among Dutch construction workers. Scand J Work Environ Health.

[ref5] Alavinia S. M, de Boer A. G, van Duivenbooden J. C, Frings-Dresen M. H, Burdorf A (2009a). Determinants of work ability and its predictive value for disability. Occup Med (Lond).

[ref6] Alavinia S. M, van den Berg T, van Duivenbooden C, Elders L, Burdorf A (2009b). Impact of work-related factors, lifestyle, and work ability on sickness absence among Dutch construction workers. Scand J Work Environ Health.

[ref7] Alipour A, Ghaffari M, Jensen I, Shariati B, Vingard E (2007). Reliability and validity study of Persian modified version of MUSIC (musculoskeletal intervention center) –Norrtalje questionnaire. BMC Musculoskelet Disord.

[ref8] Fischer F. M, Borges F. N, Rotenberg L, LatorreMdo R, Soares N. S, Rosa P. L, Landsbergis P (2006). Work ability of health care shift workers: What matters?. Chronobiol Int.

[ref9] Ghaddar A, Ronda E, Nolasco A (2011). Work ability, psychosocial hazards and work experience in prison environments. Occupational Medicine.

[ref10] Ilmarinen J (2001). Aging workers. Occup Environ Med.

[ref11] Lin S, Wang Z, Wang M (2006). Work ability of workers in western China: reference data. Occup Med (Lond).

[ref12] Kaleta D, Makowiec-Dabrowska T, Jegier A (2004). Leisure-time physical activity, cardiorespiratory fitness and work ability: a study in randomly selected residents of Lódź. Int J Occup Med Environ Health.

[ref13] Kaleta D, Makowiec-Dabrowska T, Jegier A (2006). Lifestyle Index and Work Ability. International Journal of Occupational Medicine and Environmental Health.

[ref14] Martinez M. C, Mdo Latorre R (2006). Health and work abilityamong office workers. Rev Saúde Pública.

[ref15] Monteiro M. S, Ilmarinen J, Corrâa Filho H. R (2006). Work ability of workers in different age groups in a public health institution in Brazil. Int J Occup Saf Ergon.

[ref16] Pohjonen T (2001). Perceived work ability of home care workers in relation to individual andwork-related factors in different age groups. Occup Med (Lond).

[ref17] Sjögren-Rönkä T, Ojanen M. T, Leskinen E. K, Mustalampi S. T, Mälkiä E. A (2002). Physical and psychosocial prerequisites of functioning in relation to work ability and general subjective well-being among office workers. Scand J Work Environ Health.

[ref18] Stattin M (2005). Retirement on grounds of ill health. Occup Environ Med.

[ref19] Tuomi K, Luostarinen T, Ilmarinen J, Klockars M (1991). Work load and individual factors affectingwork ability among aging municipal employees. Scand J Work Environ Health.

[ref20] Tuomi K, Ilmarinen J, Jahkola A, Katajarinne L, Tulkki A (1994). Work Ability Index. Occupational Health Care.

[ref21] Tuomi K, Ilmarinen J, Martikainen R, Aalto L, Klockars M (1997). Aging, work, life-style and work abilityamong Finnish municipal workers in 1981–1992. Scand J Work Environ Health.

[ref22] Tuomi K, Huuhtanen P, Nykyri E, Ilmarinen J (2001). Promotion of work ability, the quality of work and retirement. Occup Med (Lond).

[ref23] Tuomi K, Vanhala S, Nykyri E, Janhonen M (2004). Organizational practices, work demands and thewell-being of employees: a follow-up study in the metal industry and retail trade. Occup Med (Lond).

[ref24] van den Berg T, Elders L, de Zwart B, Burdorf A (2009). The effects of work-related and individual factors on the Work Ability Index: a systematic review. Occup Environ Med.

